# Huge Trichilemmal Carcinoma With Metastasis Presenting With Two Distinct Histological Morphologies: A Case Report

**DOI:** 10.3389/fonc.2021.681197

**Published:** 2021-09-06

**Authors:** Yao Xie, Lin Wang, Tingting Wang

**Affiliations:** Department of Dermatovenerology, West China Hospital, Sichuan University, Chengdu, China

**Keywords:** trichilemmal carcinoma, distant metastasis, histological morphologies, case report, skin tumor

## Abstract

**Background:**

Trichilemmal carcinoma (TC) is a rare malignancy of cutaneous adnexal carcinoma, with only 136 cases reported in the literature to date. It usually has an indolent course and benign clinical evolution, and only four cases with regional and distant metastasis have been reported. Tumor cells present with the characteristics of trichilemmal differentiation on both histological and immunohistological examination.

**Case Presentation:**

We report a case of TC on the scalp with an aggressive course and metastasis to the ipsilateral neck. Moreover, the lesions presented with two distinct histological morphologies.

**Conclusion:**

Despite an indolent course and benign clinical evolution, TC has the potential for local invasion and recurrence, which implies that accurate early diagnosis and careful follow-up are very important for these patients. More than one specimen should be obtained for histopathological examinations when the lesion is very large and characterized by different morphologies.

## Introduction

Trichilemmal carcinoma (TC) is a rare malignant tumor that is usually less than 3 cm. TC with metastasis is extremely rare. Headington first proposed the term “trichilemmal carcinoma” based on its histopathology, exhibiting features of “histological invasion, cytologically atypical clear cell neoplasm of adnexal keratinocytes which is in continuity with the epidermis and/or follicular epithelium”. (1) Herein, we report a very interesting case of TC in which the lesion was more than 10 cm with metastasis. Moreover, this is the first case of TC presenting with two different morphologies on histopathological examinations. Hence, additional studies are needed to further understand this rare tumor. Despite an indolent course and benign clinical evolution, this tumor has the potential for local invasion and recurrence, which implies that both accurate early diagnosis and careful follow-up are very important.

## Clinical Data

**Figure 1:**
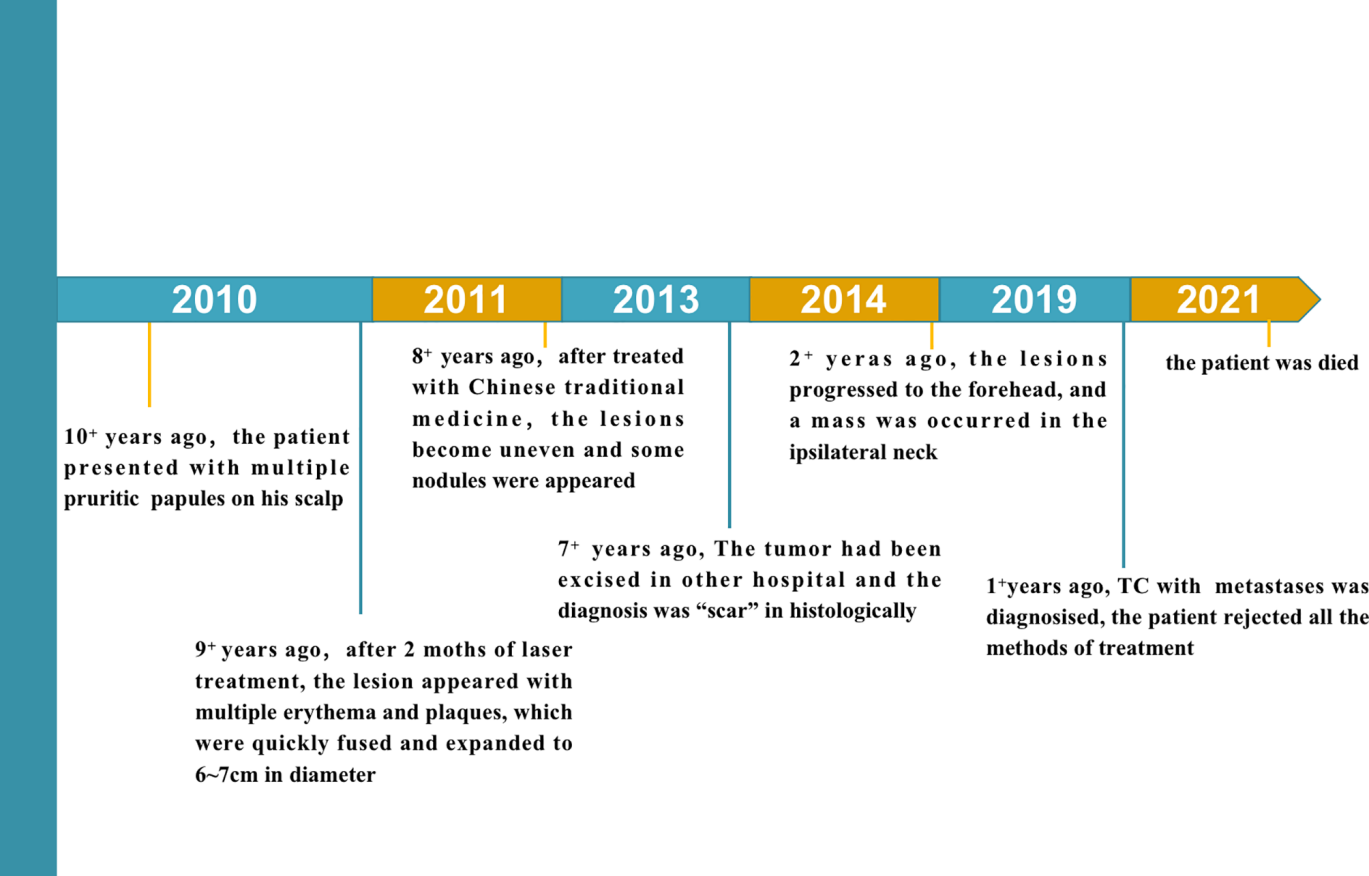
The medical history of the patient.

A 64-year-old man presented with a history of multiple asymptomatic plaques and nodules on his scalp for 9 years. He reported that multiple pruritic papules had first been observed 9 years earlier. The papules and plaques gradually grew to approximately 6 cm × 7 cm in size. The tumor was excised at another hospital in 2014, and the diagnosis was “scar”. Seven months before, multiple nodules had formed on the scalp, and the lesion rapidly invaded the left forehead. Moreover, a mass was also observed on the ipsilateral neck (**Figure 1**). There were no systemic symptoms or other underlying diseases, including skin disorders. He had no family history of the disease.

**Figure 2:**
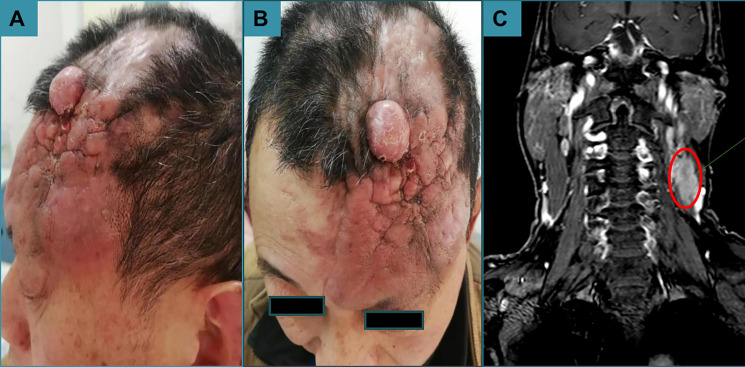
Dermatological examination. **(A)** A reddish, tender mass 12 cm × 10 cm in size was observed. **(B)** An ulceration 1 cm × 1 cm in size was found in the center of the mass. **(C)** The forehead was unevenly thickened and hardened, and there was an irregular soft tissue shadow on the left posterior cervical area that may have been neoplastic lesions.

Physical examination revealed multiple interfused reddish, tender plaques and nodules, which were 12 cm × 10 cm in size on his scalp (**Figure 2A**). The lesions had a 1 cm × 1 cm ulceration in the center (**Figure 2B**). Moreover, there was a skin-colored mass on the ipsilateral neck, which was 3 cm × 3 cm in size. The systematic physical examination showed no abnormalities. Laboratory tests including blood cell counts, urea, and liver function were normal. Computed tomography of the chest and abdomen was also normal. Magnetic resonance imaging found that the forehead was unevenly thickened and hardened, and there was an irregular soft tissue shadow on the left posterior cervical area that may have been neoplastic lesions (**Figure 2C**). Incisional biopsies of a nodule and a plaque on the left scalp and an incisional biopsy of the mass on the ipsilateral neck were taken and sent for histopathological examination.

**Figure 3:**
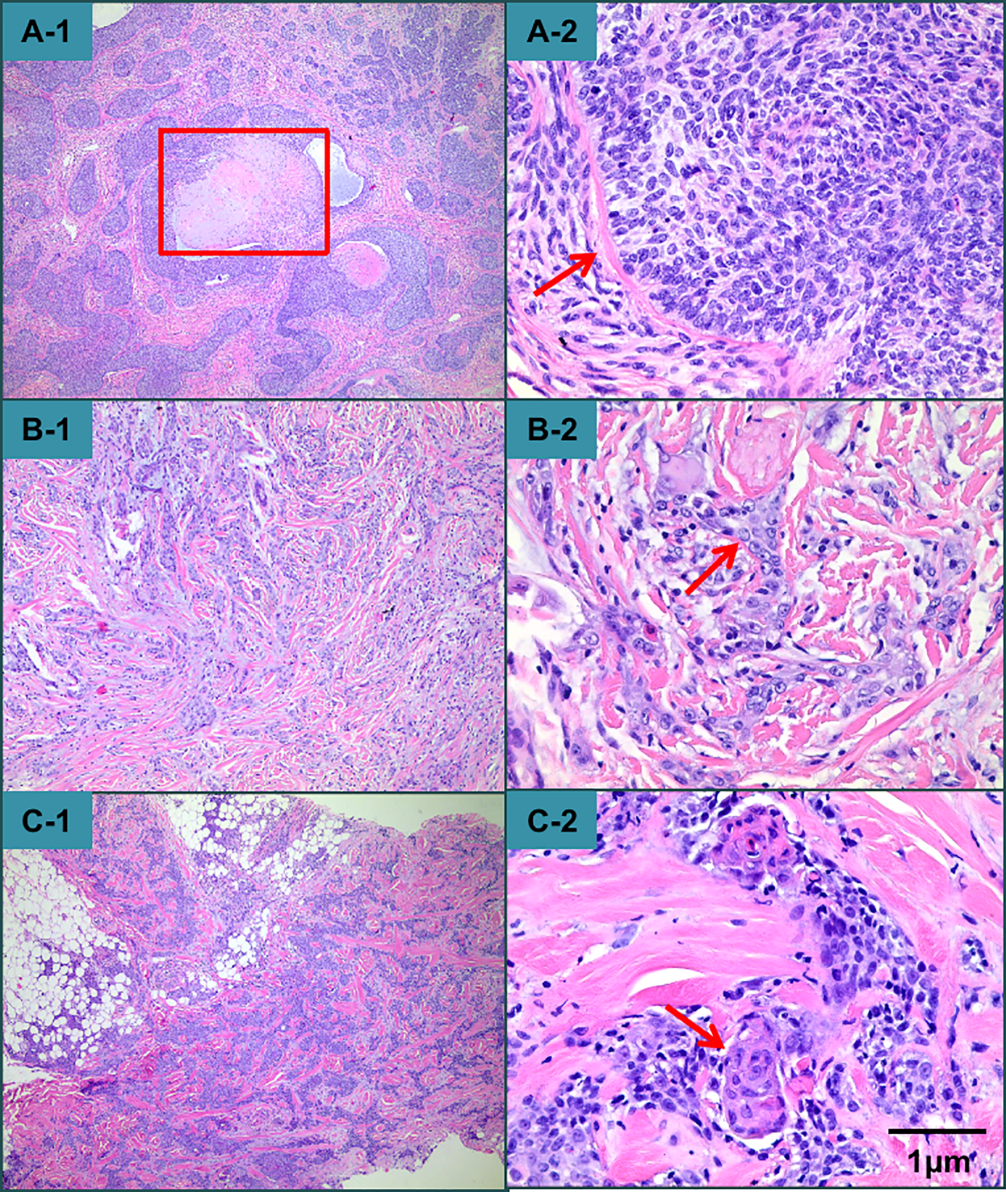
Histopathological examination revealed that the nodule exhibited epithelial tumor islands interspersed with a dense fibrous connective tissue stroma and centrally located trichilemmal keratinization. **(A-1)** At higher magnification, the tumor had polygonal cells, palisade arrangements with focal inversion, a thick hyaline membrane surrounding each lobule, and atypical cytology. A high mitotic index and atypical mitotic figures were observed. **(A-2)** The presentation of the plaque and the mass on the neck was distinct from that of the nodular lesion on the scalp. There were some cable-like structures, which may have even reached from the dermis to the subcutaneous fat in the mass on the neck **(B-1, B-2, C-1, C-2)**.

**Figure 4:**
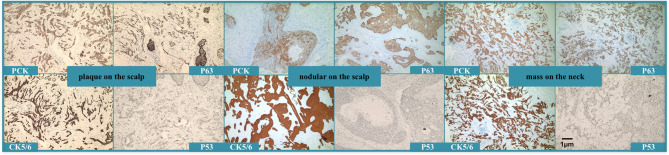
Immunohistochemical staining showed that all the lesions expressed PCK, CK5/6, P63, and P53, but was Ber-EP4-negative. The plaque on the scalp and the mass on the neck also showed CAM5.2, CK7, CEA, and EMA were negative, and S-100 was negative in the nodule of the scalp and the mass of the neck. CD34 was only expressed in nodules on the scalp and was negative in plaques on the scalp and masses on the neck.

Histologically, the nodule was a classic presentation of TC, whereas the plaque and the mass on the neck presented with some cable-like structures, which looked like sweat glands in the dermis of the lesion and may even have invaded from the dermis to the subcutaneous fat (**Figure 3**). However, the immunohistochemical results of all the specimens were consistent with trichilemmal differentiation (**Figure 4**). Therefore, the final diagnosis was TC with metastasis.

## Discussion

Cutaneous adnexal carcinomas are rare and reported to represent 0.005% of all skin tumors. These carcinomas have a heterogeneous origin and originate from undifferentiated stem cells (2). TC clinically appears as a papule, plaque, or nodule, which mostly occurs in sun-exposed areas of older people. Normally, the size of TC is less than 3 cm (3). This malignant tumor is rare; to date, a total of 136 cases of TC have been reported (4–10). It usually has an indolent course and benign clinical evolution, and only four cases with regional and distant metastasis have been reported (11–14). The most frequent metastatic organ was regional lymph nodes. In addition, all the patients with metastasis died. Hence, once metastasis occurs, the prognosis is very poor.

Histologically, TC exhibits a lobular arrangement, peripherally arranged hyperchromatic cells, centrally located glycogen-rich periodic acid–Schiff (PAS)-positive clear cells, and a thickened basement membrane. Pleomorphism and mitotic activity of tumor cells were also found (15). Interestingly, our patient presented with two distinct histological morphologies. The nodular lesion on the scalp fulfilled the criteria for TC, but there were some cable-like structures resembling sweat glands in the scalp and neck lesions. To the best of our knowledge, this is the first case of TC exhibiting two different morphologies based on histopathological examination. Although TC generally has a benign clinical course, regional or distant metastasis can occur. Our patient presented with ipsilateral neck metastasis. Unfortunately, the optimal treatment for this neoplasm occurring with metastasis has not yet been determined, and there is no established chemotherapy regimen to reference in the literature (11). Thus, it is very important to make an accurate diagnosis at an early stage. More than one specimen should be obtained for histopathological examination when the lesion is very large and characterized by different morphologies. Furthermore, we think that immunochemical examination would be as valuable as histopathological examination since TC can resemble basal cell carcinoma (BCC) or squamous cell carcinoma (SCC) both clinically and histopathologically. Histopathological evaluation alone may lead to misdiagnosis. TC cells usually exhibit increased proliferative activity and are Ber-EP4-negative, unlike BCCs, which are usually Ber-EP4-positive and lack hair follicle differentiation features (5). We distinguished this case of TC from SCC by hematoxylin and eosin and PAS staining patterns. Unfortunately, the patient refused all treatments and died in April 2021.

We herein report a very interesting case of TC, as this is a rare malignant tumor that is usually less than 3 cm; TC with metastasis is extremely rare, and the lesion in our case was more than 10 cm with metastasis. Moreover, this is the first case of TC presenting with two different morphologies on histopathological examination; more studies are needed. Despite an indolent course and benign clinical evolution, this tumor has the potential for local invasion and recurrence, so accurate early diagnosis and careful follow-up are very important for these patients.

## Data Availability Statement

The original contributions presented in the study are included in the article/supplementary material. Further inquiries can be directed to the corresponding author.

## Ethics Statement

Written informed consent was obtained from the individual(s) for the publication of any potentially identifiable images or data included in this article.

## Author Contributions

YX collected and reviewed the literature and wrote the manuscript. YX wrote and revised the manuscript. LW rechecked the manuscript and put forward meaningful comments on it. TW contributed to writing design and revised the manuscript. All authors contributed to the article and approved the submitted version.

## Funding

This work was supported by the Scientific and Technological Office of Sichuan Province (grant numbers 2019YFS0250 and 2020YFS0196).

## Conflict of Interest

The authors declare that the research was conducted in the absence of any commercial or financial relationships that could be construed as a potential conflict of interest.

## Publisher’s Note

All claims expressed in this article are solely those of the authors and do not necessarily represent those of their affiliated organizations, or those of the publisher, the editors and the reviewers. Any product that may be evaluated in this article, or claim that may be made by its manufacturer, is not guaranteed or endorsed by the publisher.
